# Different pitcher shapes and trapping syndromes explain resource partitioning in *Nepenthes* species

**DOI:** 10.1002/ece3.1920

**Published:** 2016-02-03

**Authors:** Laurence Gaume, Vincent Bazile, Maïlis Huguin, Vincent Bonhomme

**Affiliations:** ^1^ Université de MontpellierUMR AMAP: botAnique et Modélisation de l'Architecture des Plantes et des végétationsCIRAD – TA A51/PS2Boulevard de la LirondeF‐34398MontpellierFrance; ^2^ CNRSUMR AMAP: botAnique et Modélisation de l'Architecture des Plantes et des végétationsCIRAD – TA A51/PS2Boulevard de la LirondeF‐34398MontpellierFrance; ^3^ Université François Rabelais60 rue du Plat D'EtainF‐37020ToursFrance; ^4^ Université de MontpellierUMR 5554 Institut des Sciences de l'EvolutionPlace Eugène BataillonF‐34095MontpellierFrance

**Keywords:** Adaptation, carnivorous plant, niche segregation, pitcher morphology, prey composition

## Abstract

*Nepenthes* pitcher plants display interspecific diversity in pitcher form and diets. This species‐rich genus might be a conspicuous candidate for an adaptive radiation. However, the pitcher traits of different species have never been quantified in a comparative study, nor have their possible adaptations to the resources they exploit been tested. In this study, we compare the pitcher features and prey composition of the seven *Nepenthes* taxa that grow in the heath forest of Brunei (Borneo) and investigate whether these species display different trapping syndromes that target different prey. The *Nepenthes* species are shown to display species‐specific combinations of pitcher shapes, volumes, rewards, attraction and capture traits, and different degrees of ontogenetic pitcher dimorphism. The prey spectra also differ among plant species and between ontogenetic morphotypes in their combinations of ants, flying insects, termites, and noninsect guilds. According to a discriminant analysis, the *Nepenthes* species collected at the same site differ significantly in prey abundance and composition at the level of order, showing niche segregation but with varying degrees of niche overlap according to pairwise species comparisons. Weakly carnivorous species are first characterized by an absence of attractive traits. Generalist carnivorous species have a sweet odor, a wide pitcher aperture, and an acidic pitcher fluid. Guild specializations are explained by different combinations of morpho‐functional traits. Ant captures increase with extrafloral nectar, fluid acidity, and slippery waxy walls. Termite captures increase with narrowness of pitchers, presence of a rim of edible trichomes, and symbiotic association with ants. The abundance of flying insects is primarily correlated with pitcher conicity, pitcher aperture diameter, and odor presence. Such species‐specific syndromes favoring resource partitioning may result from local character displacement by competition and/or previous adaptations to geographically distinct environments.

## Introduction

One of the fundamental aims of research in ecology and evolution is to understand the origin and cause of species diversity. Adaptive radiations are a major feature of species diversification. An adaptive radiation is defined as the rapid diversification of a lineage into species displaying different morphological or physiological traits used to exploit a variety of different resources (Schluter 2000). Archipelagos often favor adaptive radiations because the different islands represent different opportunities for colonization and habitats characterized by specific climatic and ecological conditions (Jorgensen and Olesen 2001). The best known adaptive radiation is certainly that of Darwin's finches in the Galapagos Islands. This example presents the evolution of different species of the Geospizinae with specific morphological adaptations of their beak to different diets reflecting the resources of their habitat (Darwin 1859; Grant and Grant 2002).

Similarly, the carnivorous plants of the genus *Nepenthes* (Caryophyllales: Nepenthaceae) may be good candidates for an adaptive radiation with respect to trap morphology and nutrient sequestration strategy (Pavlovič 2012). The genus *Nepenthes* is distributed from Madagascar to New Caledonia and comprises 164 species, with hotspots of diversity in the islands of Borneo (Clarke 1997), Sumatra (Clarke 2001), and the Philippines (McPherson 2009). The islands of Southeastern Asia have undergone an eventful geological and climatic history during the most recent glaciations with numerous episodes of sea‐level changes, creating refuge zones for species and block settlements (Woodruff 2010). Some studies based on fossil data give an estimate of the first diversification of *Nepenthes* at 65 million years, with a diversification in South‐Eastern Asia dating from the Pliocene/Pleistocene, that is, 1.5–3 million years (Meimberg et al. 2001). The genus *Nepenthes*, with such a relatively recent diversification and with its numerous species, most of which are endemic species with restricted geographical distributions, could thus represent a conspicuous case of rapid and profuse speciation. However, neither the interspecific diversity in trap morphology nor its possible adaptive significance has so far been investigated based on quantitative approaches.

The *Nepenthes* species are tropical vines that grow solely or coexist in different habitats such as heath forest, peat swamp forest, mangroves, and cloudy montane forests on diverse infertile substrates including white sands, peat, cliffs, ultramafic soils, or epiphytic substrates. All these substrates are characterized by a scarcity of nutrients, especially nitrogen, or their nonavailability in a form easily assimilated (Vitousek and Howarth 1991; Yule 2010). These vines have evolved, at the apex of their leaf tendrils, traps in the form of fluid‐filled pitchers that most often capture small arthropods. These arthropods represent a major portion of the plant's nutrient budget (Schulze et al. 1997; Moran et al. 2001; Bazile et al. 2012). The *Nepenthes* vines exhibit interspecific diversity in the size and form of the trap, which may be narrow or funnel‐shaped (Gaume and Di Giusto 2009), ovoid (Cresswell 1998), exceptionally large (Chin et al. 2010) or dome‐shaped (Moran et al. 2012). They also exhibit intraspecific diversity in the form of the trap and often produce aerial pitchers that differ morphologically from terrestrial ones. Indeed, like most vines, *Nepenthes* pitcher plants are heteroblastic species characterized by leaf ontogenetic dimorphism, which is well marked in certain species (Gaume and Di Giusto 2009). Such a change in leaf form is often associated with the transition from a juvenile stage to a mature, flower‐producing stage (Lee and Richards 1991). The genus *Nepenthes* also displays a great diversity of mechanisms of insect attraction and capture (Moran et al. 1999, 2013; Bonhomme et al. 2011b; Bazile et al. 2015). To attract insect prey, *Nepenthes* species display different attracting signals ranging from nectar rewards (Bauer et al. 2009), emission of volatile compounds (Di Giusto et al. 2010) to visual cues (Moran et al. 1999). To capture insects, the genus deploys different combinations of mechanisms as diverse as a wettable peristome (Bohn and Federle 2004), trap walls covered by a slippery waxy layer (Gaume et al. 2002), traps filled with a viscoelastic digestive fluid (Gaume and Forterre 2007), or light traps (Moran et al. 2012).

Additionally, there is also an increasing body of evidence that *Nepenthes* pitcher plants differ in their diet, with certain species displaying nutrition strategies, which are sometimes more detritivorous (Moran et al. 2003; Pavlovič et al. 2011) or partially coprophagous (Clarke et al. 2009; Grafe et al. 2011) than purely carnivorous strategies. Among insectivorous plants, although ants and flies are the two main prey items, the prey assemblages appear to differ among *Nepenthes* species (Kato et al. 1993; Adam 1997; Chin et al. 2014).

However, there is still little evidence in the genus *Nepenthes* for any adaptive significance of trap characteristics in terms of species' diet. The possible correspondence between trap geometry and type of nitrogen source has only been investigated in the noncarnivorous species bearing large traps, such as *Nepenthes rajah*,* N. lowii,* and *N. macrophylla*. For those species, the trap geometry perfectly matches the body size of the tree shrew that defecates into the trap (Chin et al. 2010). In carnivorous species, some studies have shown that specific traits can favor the capture of a specific guild of insects. In this way, the capture of flies is favored by viscoelastic fluids (Bonhomme et al. 2011b; Bazile et al. 2015) or translucent tissues acting as light traps (Moran et al. 2012), while slippery waxy walls are sufficient against ants (Bonhomme et al. 2011b). However, no attempt has yet been made to disentangle and test the role of the trap form. Form and trapping features can, however, be linked. In *Nepenthes rafflesiana*, for example, the lower terrestrial pitchers borne by the self‐supporting plants are typically large at their base and narrower in their upper part, while the aerial pitchers in the climbing plants are funnel‐shaped. In this species, the pitcher dimorphism is coupled with a loss of the waxy zone (Gaume and Di Giusto 2009), an enhancement of fluid viscosity and a change in prey composition (Di Giusto et al. 2008).

**Figure 1:**
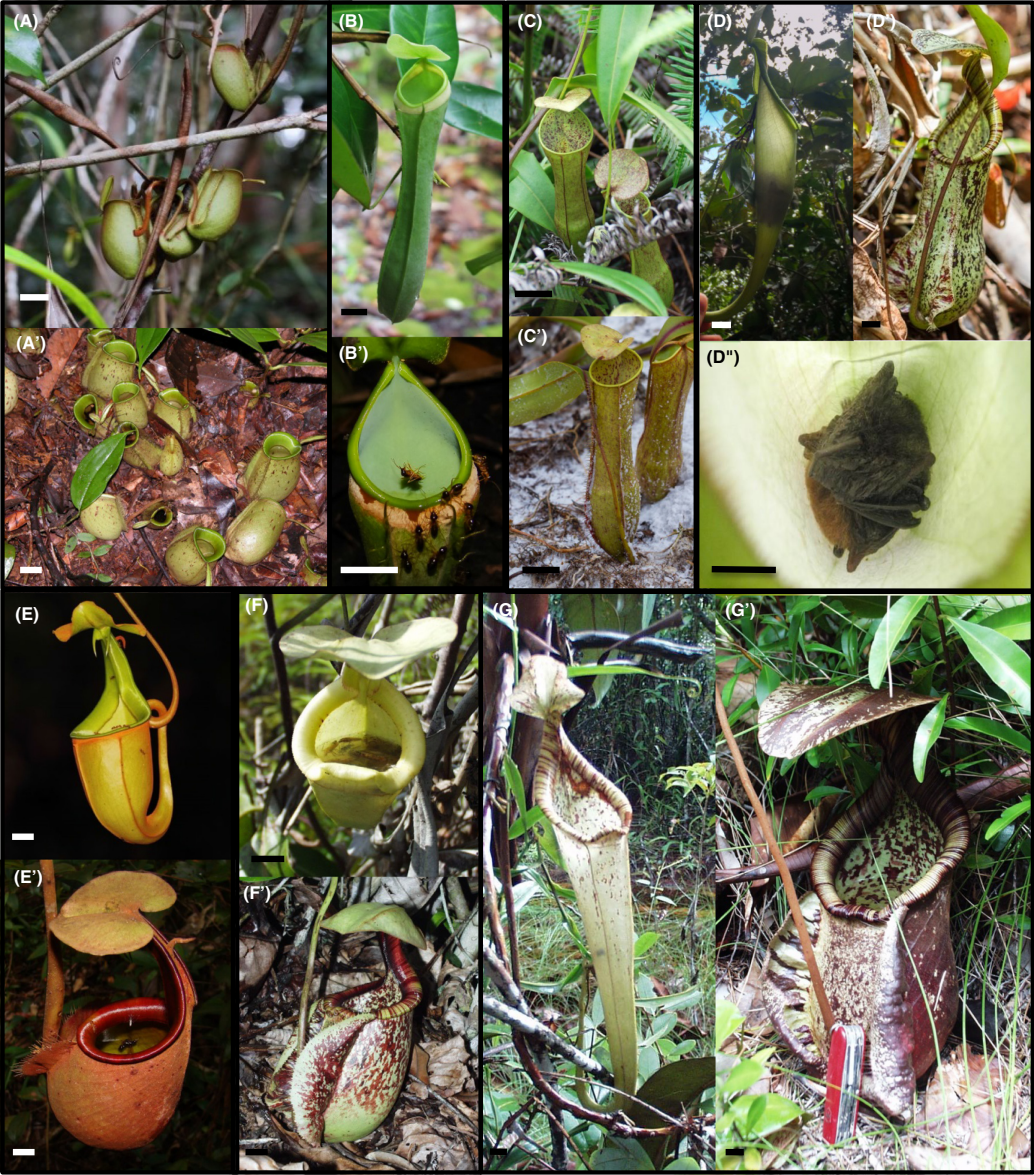
Upper (I) and lower (I′) pitchers of the seven *Nepenthes* taxa used in the study. (A) *N. ampullaria* known to use vegetal detritus as main food source. Note that in this species, the aerial pitcher shown (A) is not the true morphotype of the upper pitcher characterizing the lianescent stage of the vine, which is rarely exhibited in this species because the species remains for a long period in the self‐supporting stage. (B) *N. albomarginata* displays a rim of white edible trichomes that attract termites en masse (B′). (C) *N. gracilis*. (D) *N. hemsleyana,* known to use the feces of the bat, *Kerivoula hardwickii (*D″*)* as a complementary food source. (E) *N. bicalcatata*, the myrmecophyte harboring the ant *Camponotus schmitzi*. (F) *Nepenthes rafflesiana* var. *typica*. (G) *N. r*. var. *gigantea*. The scale bars represent 1 cm.

The goal of this study was to test for a correlation between plants' pitcher form, trapping feature, and prey composition in lowland *Nepenthes* species of Northern Borneo, to assess the niche differentiation of those species and to discuss the extent to which morphological differentiation in the genus *Nepenthes* has driven niche differentiation and allowed species coexistence. The study was conducted in the lowland forests of Brunei and focused on seven *Nepenthes* taxa including six true species, namely *N. albomarginata*,* N. ampullaria*,* N. bicalcarata*,* N. gracilis*,* N. hemsleyana*,* N. rafflesiana* var. *typica*, and *N. rafflesiana* var. *gigantea* ined. (Fig. 1). We attempted to respond to the following three questions. Do the species differ in their trap morphology and combination of trapping features, including those involved in the attraction, capture and retention system? Do they differ in prey composition when growing at the same site? If yes, how may the different trap characteristics contribute to such a niche differentiation?

## Materials and Methods

The study was conducted in the heath forest located along the Labi road of Brunei at two distinct sites separated by a dozen kilometers (site 1 located at 4°44′N, 114°35′E, site 2 located at 4°56′N, 114°49′E). All the species except *N. albomarginata* were found in August 2009 at site 1; *N. albomarginata* was studied in August 2012 at site 2 where the other species, except *N. bicalcarata*, were also present. The heath forest is the natural habitat of *N. rafflesiana*, with the giant form, further called *N. r*. var. *gigantea,* most commonly found deeper inside the forest and the typical form at its margins. *Nepenthes albomarginata* is found in the light gaps of the heath forest, as well as *N. hemsleyana*, formerly called *N. rafflesiana* var. *elongata* (Gaume and Di Giusto 2009) or *Nepenthes baramensis* (Clarke et al. 2011). *Nepenthes gracilis* is found in greater abundance at the margins of the heath forest. *N. bicalcarata*, which is closely associated with the ant *Camponotus schmitzi*, is most commonly found in the peat swamp forest, but it can also grow in the heath forest (Clarke 1997).

A total of 589 pitchers (for the detailed numbers of replicates per pitcher category and species, see Table S1) were measured for fluid pH, pitcher maximal diameter, and volume (obtained either by direct measures or from multiple‐regression extrapolations from height up to peristome, height up to opercula, and aperture maximal diameter, Table S2). All these measurements were made at the two sites during the two study periods. Because no site difference was found, the measurements were combined to make a single dataset, and a species average was calculated for each type of measure.

Three shape indexes were calculated from height up to peristome (*h*), aperture diameter (2*r*), and volumes of pitchers to measure the resemblance of pitchers to three given shapes, that is, cylinder, cone, and sphere shapes, as follows:

$$\text{cylindricity index}=\frac{|\text{pitcher volume}-h\times \pi \times r^{2}|}{\text{pitcher volume}},$$

where *h *× *π* × *r*² is the volume of a cylinder of height *h* and diameter 2*r*,

$$\text{conicity index}=\frac{\left|\text{pitcher volume}-h\times \pi \times (r^{2}/3)\right|}{\text{pitcher volume}},$$

where *h *× *π* × *r*²/3 is the volume of a cone of height *h* and diameter 2*r*, and

$$\text{sphericity index}=\frac{\left|\text{pitcher volume}-(4\times \pi \times {\left(h/2\right)}^{3})/3\right|}{\text{pitcher volume}},$$

where 4 × *π* × (*h*/2)^3^/3 is the volume of a sphere of diameter *h*.

Qualitative characteristics such as pitcher dominant color, visible presence of nectar secretion, sweet odor delivery, presence of a waxy layer on the upper inner face of the pitcher, viscoelastic behavior of the pitcher fluid, presence of a rim of white edible trichomes on the upper outer face of the pitcher, and association with a symbiotic ant species were also recorded. For all these categorical variables, a binary quantitative score was applied (0/1).

To visualize how plant species and pitcher types varied according to their combinations of plant features, a principal component analysis (PCA) was performed on the 589 pitchers based on the quantitative data collected for the plants.

A total of 70 pitchers, 10 pitchers per taxon (five terrestrial and five aerial pitchers), were analyzed for prey composition. Prey were collected in 70% ethanol, sorted, and identified up to the order level using a binocular microscope and several taxonomic guides.

To test a niche segregation of *Nepenthes* species in relation to their prey and to factor out any site effect on prey spectra difference, a discriminant analysis was performed from prey compositions on the six *Nepenthes* taxa collected in the same site (60 pitchers), thus excluding *N. albomarginata*.

As the *Nepenthes* species differed primarily in their prey combination of ants, flying insects, termites, and noninsect prey, we tested for a correlation between pitcher qualitative and quantitative characteristics (the means obtained from the first plant data set were considered) and the abundance of each of these arthropod categories for the 70 pitchers. Negative binomial regressions were used to explain the abundance of social insects such as ants or termites, while Poisson regressions were used to explain the less “all or nothing” abundances of flying insects or noninsect prey. These models were, in each case, the types of model that best explained the observed variances with the smallest Akaike's information criteria. These general linear models initially tested the whole set of variables but retained only those that were significant in the tests of type 3 (i.e., when entered as last in the model). The backward selection of variables and the use of tests of type 3 allowed us to overcome any problem of multicollinearity.

All the statistical analyses were performed using the SAS v. 9.3 package (SAS Institute Inc., Cary, NC).

## Results

### Interspecific and ontogenetic differences in trap morphology and trapping features

The seven *Nepenthes* taxa differed significantly for pitcher volume, pitcher height, and pitcher aperture diameter (Fig. 1, Table 1A, B, and C). The average volumes ranged from 10 mL in *N. gracilis* to 500 mL in *N. r*. var. *gigantea* (Table S1). The volumes of upper pitchers were significantly less important than the volumes of lower pitchers, although their height was, on average, greater. This result implies that there is a significant change in pitcher shape over ontogeny, when the vine swifts from a self‐supporting stage to a lianescent stage. Such an ontogenetic pitcher dimorphism is more or less pronounced according to species, as shown by the interaction species × pitchertype, which was significant for volume and height. For example, both volume and height were similar for the lower and upper pitchers of *N. gracilis*. But the upper pitchers were markedly longer than the lower ones for *N. hemsleyana* and the upper pitchers were significantly less in volume than the lower ones in *N. bicalcarata*. The diameter differed significantly according to species, ranging from 1.6 cm in *N. gracilis* to 7.2 cm in *N. r*. var. *gigantea*, but no significant differences were found between the pitcher ontogenetic types.

**Table 1:** Results of GLM testing for the effects of *Nepenthes* species and pitcher type (lower/upper) on pitcher morpho‐functional characteristics

Variable	df	Type III SS	Mean square	*F*	*P*
(A) Volume (*R*² = 0.54)					
Species	6	2 919 113.5	486 518.9	46.7	<0.0001
Pitchertype	1	107 795.6	107 795.6	10.3	0.0014
Species × pitchertype	6	295 715.3	49 285.9	4.7	0.0001
(B) Height (*R*² = 0.66)					
Species	6	2341.5	390. 3	112.8	<0.0001
Pitchertype	1	528.8	528.8	152.9	<0.0001
Species × pitchertype	6	546.6	91.1	26.3	<0.0001
(C) Diameter (*R*² = 0.72)					
Species	6	623.9	103.9	144.9	<0.0001
Pitchertype	1	0.1	0.1	0.2	0.6669
Species × pitchertype	6	16.18	2.7	3.8	0.0011
(D) Fluid pH (*R*² = 0.77)					
Species	6	413.7	69.0	168.9	<0.0001

The fluid pH differed significantly according to species but not according to pitcher type (Table 1D). Both the myrmecophyte *N. bicalcarata* and the detritivorous species *N. ampullaria* had the lowest fluid pH, close to 5, while *N. rafflesiana* and *N. gracilis* had the most acidic fluids, with a pH close to 2 (Table S1).

The *Nepenthes* species also differed in their attractive features. Nectar secretions were visible only in *N. rafflesiana*,* N. r*. var. *gigantea*,* N. gracilis,* and *N. bicalcarata*. All these species, except *N. gracilis*, produced a sweet odor. Their pitchers displayed red or yellow colors, while the dominant color of *N. ampullaria*,* N. albomarginata,* and *N. hemsleyana*, associated with no visible nectar secretions, was green. *N. albomarginata* was the only species to display a rim of white edible trichomes at the upper outer side of its pitchers (Fig. 1B). The species also differed in their trapping features. The pitchers of some species such as *N. gracilis* and *N. albomarginata* bore a slippery waxy zone but contained waterlike fluids; some taxa, such as *N. rafflesiana* and *N. r*. var. *gigantea*, had a viscoelastic retentive fluid, as shown by its tendency to form viscoelastic filaments when stretched between the fingers. Among both taxa, only the lower pitchers of *N. rafflesiana* possessed a waxy zone. *N. hemsleyana* was the only species to have both a viscoelastic fluid and a waxy zone. *N. bicalcarata* and *N. ampullaria* were devoid of these features, but *N. bicalcarata* was symbiotically associated with the ant *C. schmitzi,* which hunts and helps the plant to catch its prey (Bonhomme et al. 2011a). All the pitchers of these species possessed a corrugated rim outlining their aperture, called the peristome, which may be highly wettable and cause insect aquaplaning (Bohn and Federle 2004).

**Figure 2:**
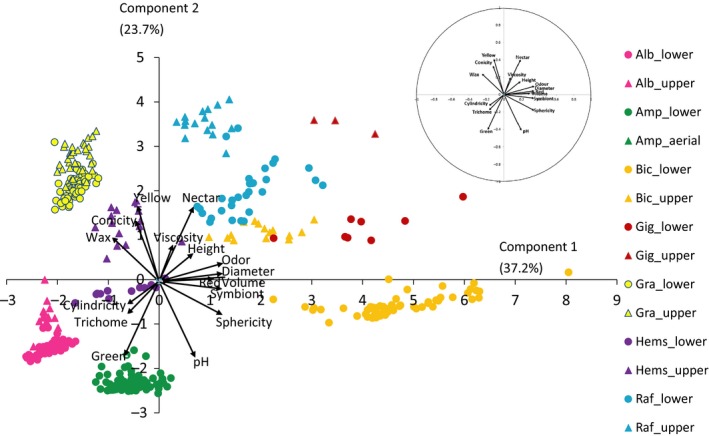
Results of a principal component analysis (PCA) analysis showing how *Nepenthes* species differ in morphology, the importance of their pitcher dimorphism, and how each morphotype reflects a specific combination of trapping features. To correctly visualize the correlations between the plant traits and the species, the eigenvectors shown in the correlation circle in the upper right corner of the graph were increased, the slopes and proportions were preserved, and the results were superposed on the PCA graph.

Interestingly, as shown by the PCA projection (Fig. 2), the *Nepenthes* species were clearly differentiated from each other by the combination of their trapping features and the degree of their pitcher dimorphism. The first two components explained 61% of the variance. The first axis, which explained 37% of the variance, separated odoriferous and voluminous pitchers with large diameters from scentless and narrow ones, while the second axis primarily segregated pitchers according to the conicity and the pH of their fluids, separating conic, mainly nectariferous pitchers of acidic fluids from others. Nevertheless, some syndromes were clearly exhibited. For example, slender pitchers were typically associated with a waxy zone, a narrow aperture and an acidic pH, while funnel‐shaped pitchers, that is, those with both a conic form and a wide aperture diameter, were typically odoriferous and nectariferous pitchers with a viscoelastic and acidic fluid. The pitchers with weakly acidic fluids had a rather rounded shape; they were also associated neither with a waxy layer nor with a viscoelastic fluid.

Moreover, the graph also shows that upper pitchers were usually more conical than lower ones and that such a pitcher dimorphism was most pronounced for *N. rafflesiana*,* N. r*. var. *gigantea,* and *N. hemsleyana*, the three taxa bearing a viscoelastic fluid.

### Interspecific and ontogenetic differences in prey abundance and diversity

**Figure 3:**
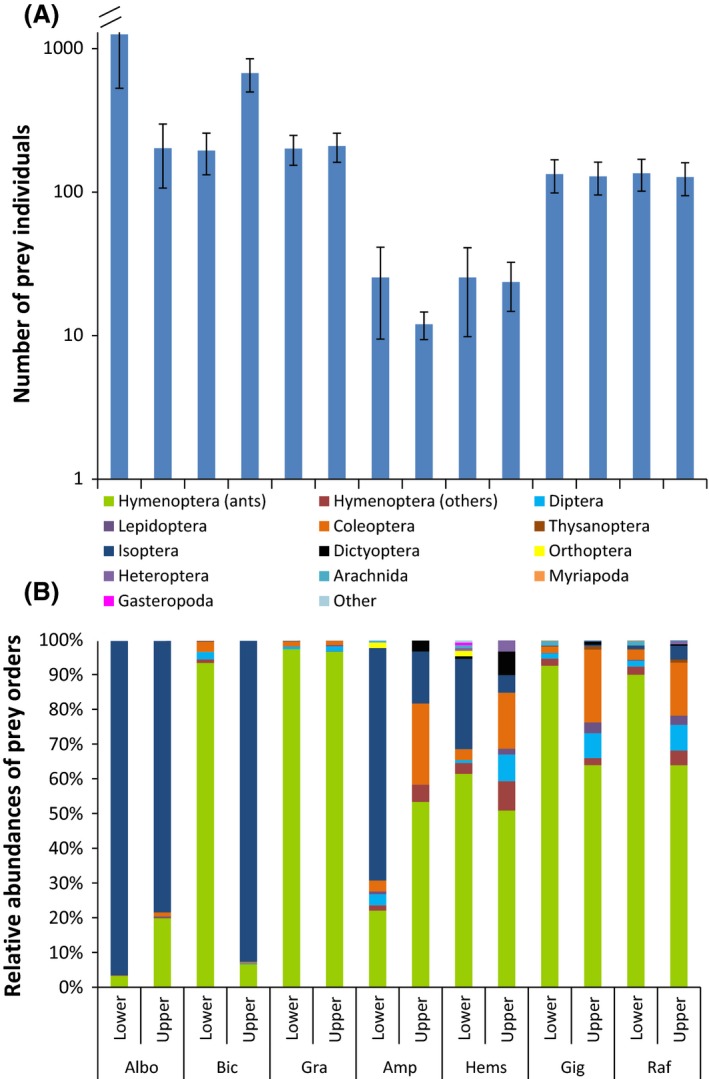
Prey abundance (A) and diversity (B) differ according to *Nepenthes* species and pitcher type.

The seven *Nepenthes* taxa differed significantly in prey abundance (Table 2A), with *N. albomarginata* bearing the most efficient pitchers, containing as many as 1000 prey individuals and more (Table S1). This species was closely followed in total pitcher content by *N. bicalcarata*. At the opposite extreme are *N. hemsleyana* and *N. ampullaria*, which barely reached 25 prey items per pitcher. There was no effect of pitcher type, but a significant effect of pitcher type × species interaction on prey abundance implied that for certain species, upper pitchers were more efficient than lower ones, whereas the opposite was the case for other species (Fig. 3A and Tables 2A and S1).

The *Nepenthes* species also differed in the diversity of animal prey trapped in their pitchers. Prey order richness differed significantly by *Nepenthes* species (Fig. 3B, Table 2B) but not significantly according to pitcher type: (*χ*² = 2.25, df = 1, *P *=* *0.13). *N. rafflesiana* and *N. r*. var. *gigantea* were the most generalist species (Fig. 3B, Table S1). They trapped up to nine orders, most of which were flying insects, for example, Coleoptera, Diptera, Hymenoptera, Lepidoptera, and Thysanoptera. *N. albomarginata* and *N. gracilis* were the most specialized species, the former trapping primarily termites and the latter several species of ants (Fig. 3B). The ants (Formicidae) found in the *Nepenthes* traps belonged to subfamilies Formicinae, Myrmicinae, Dolichoderinae, and Pseudomyrmicinae. They were all wingless workers, except three “alate” founding queens (two of *C. schmitzi* found in two traps of *N. bicalcarata* and one of *Tetraponera* sp. found in an upper pitcher of *N. rafflesiana)*. The termites found in *Nepenthes* traps were all wingless workers and soldiers of the genus *Hospitalitermes* (*Nasutitermitinae*,* Termitidae*) with the exception of two unidentified alates found in two traps of *N. rafflesiana*. Ants, termites, and flying insects were the three main guilds of insects trapped in the pitchers of *Nepenthes*. Noninsect orders represented a small proportion of the total prey (<5%, Fig. 3B) but probably a more considerable proportion of the total biomass. They included primarily Arachnida (the vast majority of which were spiders). Gastropoda and Myriapoda were found in greater proportions in *N. hemsleyana*,* N. rafflesiana,* and *N. r*. var. *gigantea*.

**Table 2:** Results of multiple regression tests for the effects of *Nepenthes* species and pitcher type (lower/upper) on prey richness and insect guild abundances composing pitcher prey

Type 3 Wald test			
Variable	df	*χ2*	*P*
(A) Number of prey (NB reg)			
Species	6	126.5	<0.0001
Pitchertype	1	1.0	0.3159
Pitchertype × species	6	17.15	0.0087
(B) Number of prey orders (Poi reg)			
Species	6	24.1	0.0005
(C) Number of ants (NB reg)			
Species	6	117.2	<0.0001
(D) Number of flying insects (Poi reg)			
Species	6	54.5	<0.0001
Pitchertype	1	19.9	<0.0001
(E) Number of termites (NB reg)			
Species	6	122.5	<0.0001
Pitchertype	1	0.8	0.3709
Pitchertype × species	6	58.3	<0.0001

NB reg, negative binomial regression; Poi reg, Poisson regression.

The number of ants trapped in the pitchers differed significantly according to ant species (Table 2C) but not to pitcher type. *Nepenthes gracilis* and *N. bicalcarata* were the most efficient for this insect guild, while *N. hemsleyana* and *N. ampullaria* were less efficient. The number of flying insects trapped in the pitchers differed significantly according to *Nepenthes* species and pitcher types (Table 2D). Flying insects were most abundant in the traps of *N. r*. var. *gigantea*,* N. rafflesiana,* and *N. bicalcarata*. They were also systematically more abundant in upper pitchers than in lower pitchers. The number of termites also significantly differed according to *Nepenthes* species. Termites were most numerous in the traps of *N. albomarginata* and those of *N. bicalcarata* but were present in greater numbers in lower pitchers for the former and in upper pitchers for the latter (Table 2E, significant interaction species × pitchertype).

According to the discriminant analysis performed on the species found in the same site, thus excluding *N. albomarginata* from the previous data, *Nepenthes* species differed significantly in their prey composition and were significantly segregated according to two axes (axis1 explaining 47.6% of the variance, *F*_55,207_ = 2.84, *P *<* *0.0001, axis 2 explaining 34.3% of the variance *F*_40,172_ = 2.12, *P *=* *0.0005). The significant discriminant variables were HymenoAntsNb, IsopteraNb, DipteraNb, NoninsectNb, HymenoFlyingNb, and HeteropteraNb, with Wilks' lambda equal to 0.58, 0.36, 0.22, 0.15, 0.12, and 0.10, respectively and *P *<* *0.0001 in each case.

**Figure 4:**
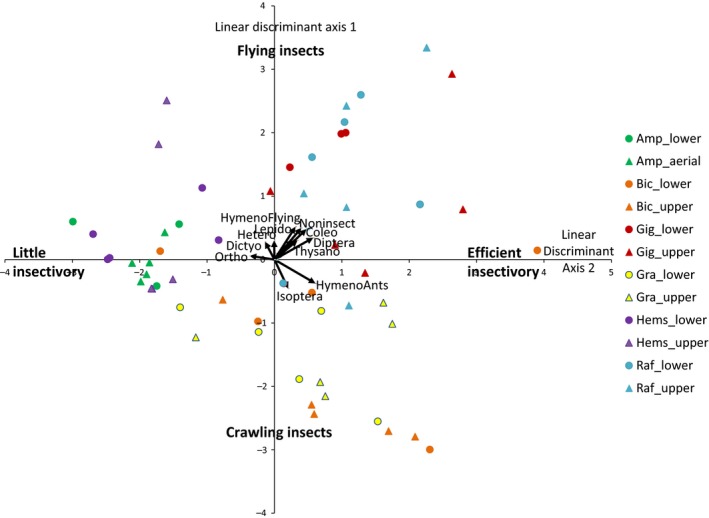
Results of a discriminant analysis showing how *Nepenthes* species differ in degree of insectivory and prey spectra. Three groups of species can be differentiated: the weakly insectivorous group (*N. ampullaria* and *N. hemsleyana*), the carnivorous group specialized on crawling and social insects (*N. gracilis* and *N. bicalcarata*), the more generalist group trapping a broad spectrum of flying insect orders (*N. rafflesiana* and *N. r*. “var. gigantea”).

The first axis separated the *Nepenthes* species according to their trapping efficiency, with low numbers of prey on the left and high numbers of prey on the right, while the second axis separated the species according to their type of prey, with crawling social insects (ants and termites) at the bottom and flying insects at the top (Fig. 4). Three groups of species clearly appeared: the poorly insectivorous group gathering *N. ampullaria* and *N. hemsleyana*, the social‐insect specialized group gathering *N. gracilis* and *N. bicalcarata* and the most generalist group including *N. rafflesiana* and *N. r*. var. *gigantea*, which trap a broader spectrum of insects including several orders of flying insects. Nevertheless, there is sometimes important pairwise niche overlap, and as a result the overall probability of misidentification of a *Nepenthes* species given a knowledge of its prey spectrum is equal to 0.38 (0.20 for *N. ampullaria*, 0.20 for *N*. *bicalcarata*, 0.60 for *N. r*. var. *gigantea*, 0.40 for *N. gracilis*, 0.10 for *N. hemsleyana*, 0.50 for *N. rafflesiana*). Interestingly, this probability decreases to 0.16 when only upper pitchers are considered (0.20 for *N. ampullaria*, 0.20 for *N. bicalcarata*, 0.00 for *N. r*. var. *gigantea*, 0.20 for *N. gracilis*, 0.40 for *N. hemsleyana*, 0.00 for *N. rafflesiana*). This finding means that upper pitchers are important determinants of niche segregation.

### Trapping features and specializations on insect guilds

**Figure 5:**
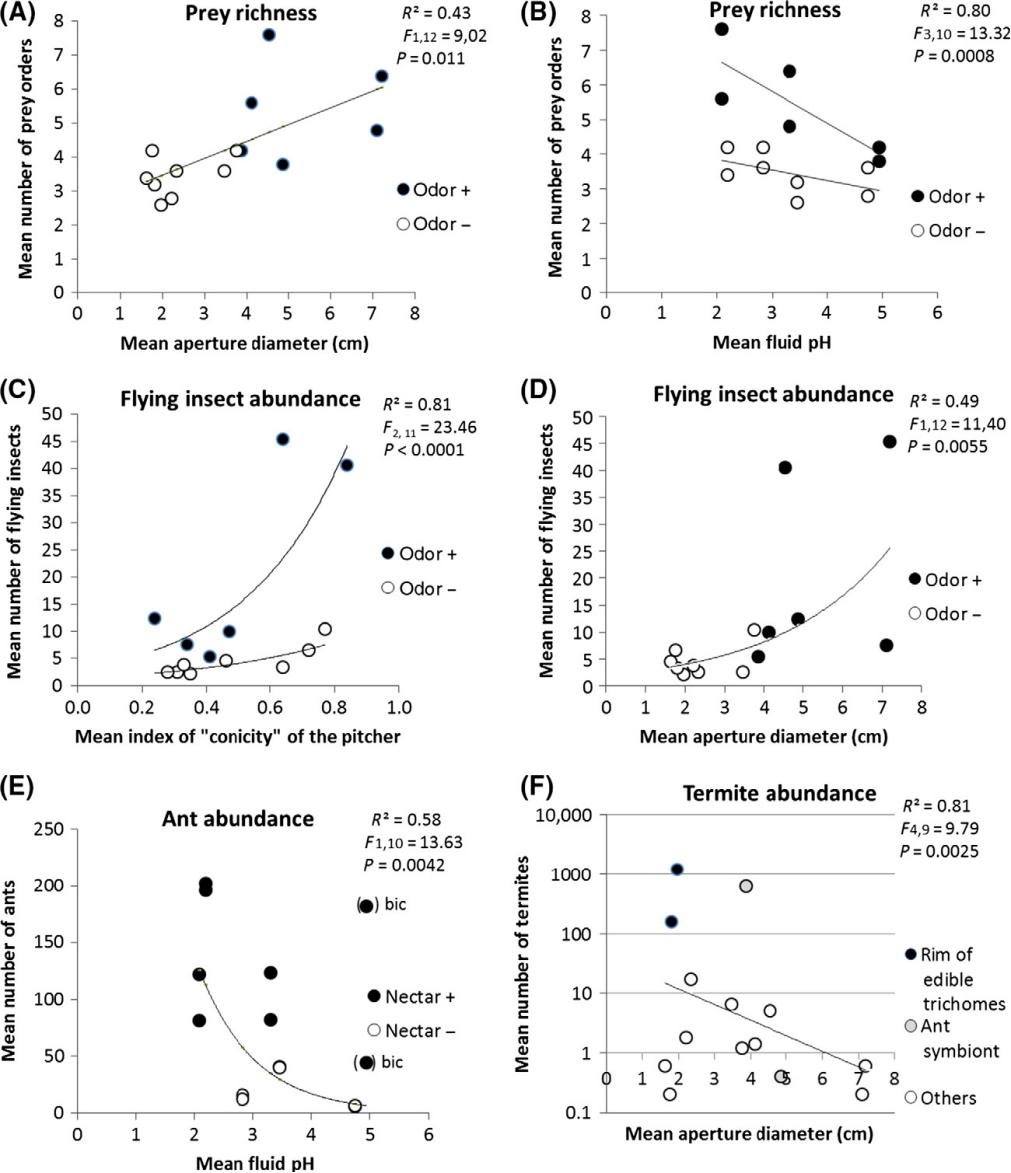
Graphical synthesis of the main correlations at the genus scale between plant features and prey richness (at the order level) or insect guild abundances. Means were computed on lower and upper pitchers of each of the seven *Nepenthes* taxa (14 dots).

Three trapping features seem to increase prey diversity in the trap of *Nepenthes*. Indeed, pH, odor, and diameter, but diameter to a lesser extent, are the variables that most effectively explained the richness of prey orders, according to a Type 3 Wald test (Poisson regression, Table 3A, Fig. 5A and B). All these variables were significant when placed in the first position in the model according to Type 1 Wald tests. Prey diversity thus decreased when the pH decreased and increased when the diameter increased. It was highest in odoriferous pitchers (lsmeans Odor_1_ = 1.61, *Z *=* *16.13, *P *<* *0.0001) and lowest in nonodoriferous ones (lsmeans Odor_0_ = 1.31, *Z *=* *16.10, *P *<* *0.0001). Figure 5 illustrates the main results shown in Table 3 but in a more concise format. The difference is that it shows only the interspecific and ontogenetic variability and is thus based on the mean insect abundances or richness (14 points corresponding to the lower and upper pitchers of each of the seven plant taxa instead of 70 points corresponding to the 70 pitchers that were analyzed).

**Table 3:** Results of the multiple regression tests for the effects of pitchers' trapping features on prey richness and arthropod guild abundance composing pitcher prey

Type 3 Wald test			
Variable	df	*χ* ^2^	*P*
(A) Number of prey orders (Poi reg)			
pH	1	6.68	0.0098
Odor	1	5.01	0.0252
Diameter	1	3.78	0.0519
(B) Number of flying insects (Poi reg)			
Conicity	1	37.27	<0.0001
Diameter	1	25.00	<0.0001
Odor	1	14.04	0.0002
(C) Number of ants (NB reg)			
pH	1	10.5	0.0012
Nectar	1	95.85	<0.0001
Wax	1	3.35	0.0692
pH × wax	1	10.3	0.0014
(D) Number of termites (NB reg)			
Diameter	1	4.7	0.0300
Ant symbiont	1	36.0	<0.0001
Trichome	1	27.4	<0.0001

NB reg, negative binomial regression; Poi reg, Poisson regression.

The number of trapped flying insects was primarily positively correlated with the degree of conicity of the pitcher, its aperture diameter, and the emission of a sweet odor (Table 3B, Fig. 5C and D). It was highest in odoriferous pitchers (lsmeans Odor_1_ = 1.65, *Z *=* *10.56 *P *<* *0.0001) and lowest in nonodoriferous ones (lsmeans Odor_0_ = 2.45, *Z *=* *15.68, *P *<* *0.0001). Fluid viscosity and fluid pH were significant variables according to Type I Wald tests, but as they were highly correlated with other variables (Fig. 2), they were no longer significant in the Type 3 test. For example, the Pearson correlation coefficients of the variable viscosity with the variables conicity, diameter, and odor were, *r* = 0.32 (*P *<* *0.007); *r* = 0.68 (*P *<* *0.0001) and *r* = 0.33 (*P *=* *0.005), respectively.

The number of trapped ants was negatively correlated with fluid pH (even more when *N. bicalcarata* is removed from the analysis, Fig. 5E) and positively correlated with nectar production and presence of a waxy inner pitcher zone (Table 3C) whereas the last relation was not significant (*P *=* *0.07, lsmeans NbAnts_waxy‐pitcher_ = 5.03, *Z *=* *18.3, *P *<* *0.0001; lsmeans NbAnts_nonwaxy‐pitcher_ = 3.07, *Z *=* *15.5, *P *<* *0.0001). Wax presence rather affected the slope of the regression linking ant number to pH (Table 3C).

The number of trapped termites was positively correlated with the presence of a white rim of edible trichomes and the presence of the symbiotic hunter ants and negatively correlated with pitcher aperture diameter (Table 3D and Fig. 5F).

## Discussion

This study, based on quantitative data, measured the differences in trap morphology in a sample of lowland *Nepenthes* species. It also measured the degree of insectivory, degree of specialization, and niche differentiation between species growing in the same site, thus factoring out any source of variability on insect prey that would have been attributable to environment. Matching both plant trapping features and prey content, our approach reveals whole trapping syndromes in relation to specific diets or nutrient sequestration strategies.

### Which features indicate that a plant should be considered carnivorous?

Our data suggest that attraction features are the most important features that condition carnivory. *Nepenthes ampullaria* and *N. hemsleyana* were prominent among other *Nepenthes* species in trapping the fewest arthropods despite efficient capture features in at least one of the two species, *N. hemsleyana* such as a slippery waxy layer and a viscoelastic fluid (Gaume and Di Giusto 2009). Both species were, incidentally, no longer considered true carnivores because they were shown to obtain a substantial amount of nitrogen from alternative sources (vegetal detritus for the former [Moran et al. 2003; Pavlovič et al. 2011] and bat feces for the latter [Grafe et al. 2011]) and may instead be classified as at least partially detritivore and coprophage, respectively. Of primary interest is that both species are also remarkable in showing no conspicuous insect‐attractive features. Their pitchers have an average green dominant color that does not stand out from the surrounding vegetation. Their peristome/body contrast of color is weak, at least for the wave bands matching insect maxima sensitivity (Moran et al. 1999; Clarke et al. 2011). The open pitchers produce weak quantities of extrafloral nectar, usually not visible to the human eye (Merbach et al. 2001; Bauer et al. 2011), and they do not bear edible trichomes. Additionally, in each of the three regression analyses explaining the abundances of the captured insect guilds, a variable linked to attraction explained most of the variance, that is, nectar for the ants, sweet scent for the flying insects, and white edible trichomes for the termites. It has recently been proposed that a plant must have at least one adaptation (i.e., active attraction, capture, and digestion) in combination with nutrient absorption to be classified as a carnivore (Pavlovič and Saganová 2015). Our results suggest that attraction is not optional but is, on the contrary, a prerequisite for the plant to be classified as a carnivore.

Our results further suggest that the plant must possess efficient weapons to catch insects, either endogenous weapons or exogenous ones such as the efficient hunter ant, *C. schmitzi*, symbiotically associated with *N. bicalcarata* (Bonhomme et al. 2011a). Among the endogenous weapons that are noteworthy is the fluid acidity, previously considered a digestive feature (but see Bazile et al. 2015). Acidity was clearly correlated with the abundance of the main prey (ants) and also marginally with the abundance of flying insects (*P *=* *0.07, results not shown), and the acidic fluids were those that possessed the richest spectra of arthropod orders. It can be argued that the low fluid pH that is correlated to insect prey abundance could simply reflect the digestive activity of the plant and might have nothing to do with the trapping function. Indeed, the functioning of the proton pumps often triggered by insect capture may favor the optimal acidic conditions for the enzymatic activity (Athauda et al. 2004) and/or the active transport of nutrient ions released by the digested prey (An et al. 2001; Moran et al. 2010). However, a set of compelling arguments suggests that the acidic pH of the fluid is primarily a cause of prey capture. Indeed, at least in *N. rafflesiana*, there is experimental evidence that prey does not trigger a pH decrease (Bauer et al. 2009). Moreover, do the different *Nepenthes* species differ in their fluid acidity even at the beginning of pitcher opening (Bazile et al. 2015), showing that their differences in fluid pH are not the result of their different efficiency at prey capture but may instead cause such a difference. Finally, an acidic pH is known to be harmful to insects (Brodin and Gransberg 1993; Harrison 2001; Bazile et al. 2015). From this point of view, it is most likely not a coincidence if *N. bicalcarata*, which is associated with the swimming ant, *C. schmitzi* (Clarke and Kitching 1995), is shown to possess the least acidic fluid among the seven studied taxa. Therefore, even though it is an important component of the digestive process, our findings suggest that fluid acidity is also a trapping weapon of the same importance as the waxy layer or the fluid viscoelasticity. However, in contrast to the last two features, it does not seem to target any particular type of prey.

### The importance of pitcher shape in niche segregation

It is noteworthy that pitcher volume was never significant as an explanatory variable of the abundance of any guild of insects. The most voluminous pitchers did not necessarily trap the largest amount of prey. For example, *N. r*. var. *gigantea*, the pitchers of which have almost the same shape than those of *N. rafflesiana*, but with an average volume ten times greater, contained similar numbers of prey as did *N. rafflesiana* and a similar diversity of prey. The shape of the pitcher matters more than its volume in terms of the guild of arthropods trapped. Indeed, although the shape of the pitcher does not seem to influence ant abundance, it critically influences the capture of flying insects and, to a lesser extent, the capture of termites. Flying insects were clearly associated with funnel‐shaped pitchers of large diameter while, in contrast, termites appeared to be more abundant in narrower aperture pitchers. One can object that the greater abundance of flying insects in the upper strata mainly explains their overall greater number in upper pitchers compared to lower ones and that the different shapes of upper pitchers, including a larger aperture diameter, on average, only play a minor role in the “targeted” capture of flying insects. However, this objection is not supported by the observation in *N. bicalcarata* that the number of flying insects was, on average, 2.5 times more numerous in lower terrestrial pitchers than in upper pitchers. Because the two pitcher types in this species did not differ from each other in their other traits, this opposite pattern rather reflects the unique tendency of this species to bear lower pitchers of greater aperture diameters than upper ones. Hence, the large size of pitcher aperture is of key importance in the capture of flying insects. What is the underlying mechanism?

Fluid‐filled pitchers with large diameters may act as reflection–polarization traps and attract, more specifically, flying insects. Sensitivity to light polarization is very common among dipterans (Horváth and Varjú 2004), the order that constitutes the most important part of the flying insects caught by *Nepenthes*. Indeed, the greater water surface exhibited by pitchers of wide aperture diameter may be an important guiding cue for these insects, especially gravid midges or mosquitoes in search of water oviposition sites (Lerner 2014). The absence of the waxy zone, which often characterizes the funnel phenotype (Gaume and Di Giusto 2009), reduces the distance between fluid level and peristome and makes the fluid more visible to insects. In contrast, the presence of the waxy zone, which characterizes narrower and more cylinder‐shaped pitchers, may select against flying insects. However, beyond shape, whole combinations of traits may explain niche segregation in the genus *Nepenthes*.

### *Nepenthes* trapping syndromes and nutrition strategies

Our results highlight the existence of three main syndromes targeting the three main insect guilds. The “flying insect syndrome” is characterized by funnel‐shaped pitchers of large diameters, with a yellow dominant color, an acidic viscoelastic fluid, nectar secretion, and the delivery of a sweet scent. While fluid‐mediated light polarization in pitchers of large aperture diameter is likely to be an important cue for flies (Horváth and Varjú 2004) and also bees (Kraft et al. 2011), the sweet scent is certainly a cue targeting, more specifically, coleopterans and lepidopterans (Di Giusto et al. 2010). The use of sticky yellow traps in the control of pests such as aphids and whiteflies (Shimoda and Honda 2013) highlights the synergic effect of the yellow color and the viscosity property in targeting small flying insects. All of these remarks argue for the adaptive significance of such a specific combination of traits in pitcher plants specialized in the capture of flying insects.

The “ant syndrome” is less specific and is characterized primarily by nectar secretion, then by fluid acidity and, to a lesser extent, a waxy trap. Actually, waxy traps do always contain ants, but significant numbers of ants can also be found in nonwaxy traps. Extrafloral nectar is a major resource explaining ant abundance in tropical forests (Davidson et al. 2003). Thus, it is hardly surprising to find that it is the main component of the “ant syndrome” in *Nepenthes*.

The “termite syndrome” is characterized by narrower pitchers and a shape that is closer to a cylinder (*r* = 0.22, *P *=* *0.06), with nonviscous fluids (*r* = −0.24, *P *=* *0.05). Termite capture is also greatly enhanced by the presence of a rim of edible trichomes or the symbiotic presence of the hunter ant, *C. schmitzi*. What can explain such a pattern? First, cylinder‐shaped traps exhibiting vertical walls should be more efficient against crawling insects than flying ones, which can use their wings to escape. Second, ants can foresee these pitfalls while blind *Hospitalitermes* soldiers cannot avoid them. Finally, while it is still necessary to analyze the nature of the edible trichomes to explain the specific attraction of such nasute termites, one can already easily understand that, for the hunter ants which inhabit *N. bicalcarata*, the blind termites accidentally fallen into the pitchers constitute a prey type that is particularly easy to attack.

Interestingly, two syndromes can be displayed by the same species over its ontogeny. This is particularly true for species exhibiting a pronounced ontogenetic dimorphism, such as *N. rafflesiana*. Indeed, its lower pitchers are nectariferous, possess a waxy zone, have a highly acidic fluid and trap mostly ants while its upper pitchers, which are funnel‐shaped, odoriferous and possess a viscoelastic fluid, trap a greater number of flying insects. Such a dichotomy in the trapping syndrome may clearly be an adaptation by the vine to exploit the different resources available in the different strata that it visits during its development. *N. bicalcarata*, which also exhibits, a marked pitcher dimorphism, also displays differences between the diets of its two types of pitchers. Upper pitchers, which are narrower and more cylindrical than lower ones (the latter having a higher “sphericity” index), trap more termites, while lower ones trap more ants. This trend was also observed by other authors (Chin et al. 2014). It is tempting to suggest that the ontogenetic change in pitcher morphology is an adaptation to the habit of *Hospitalitermes* termites of climbing the vegetation and foraging in the aerial strata during the night (Jones and Gathorne‐Hardy 1995). *Nepenthes albomarginata*, which is known to be specialized on this insect guild (Moran et al. 2001; Merbach et al. 2002), also trapped an abundant amount of termites in the terrestrial stratum. In this species, however, both pitcher types exhibit the “termite syndrome”.

### *Nepenthes* pitcher plants: a conspicuous example of an adaptive radiation?

In this paper, for the first time in *Nepenthes*, correlations between pitcher form and identity and/or the abundance of prey content are shown at the interspecific level. A previous study has reported the influence of pitcher form on the pitcher's necromass quantity at the intraspecific level in *N. ampullaria* (Cresswell 1998). Thus, it can be suggested that genetic variation in pitcher form may have important consequences not only for the diet of the pitcher plant but also for its carnivorous status. The islands of South‐Eastern Asia have undergone numerous episodes of sea‐level changes, creating new colonization sites with opportunities for founder effects and genetic drift that may fix such variation. The eventful biogeographic history of the archipelago and its high‐elevation gradient may have also promoted numerous events of spatial isolation of the populations that may have favored reproductive isolation and speciation events. The different islands and altitudinal zones are characterized by different combinations of substrate, vegetation, and climate types that represent as many different niches for *Nepenthes* species to occupy. The species that display the same trapping syndromes may, most likely, have evolved in response to the same selective pressures. Bonhomme et al. (2011b) proposed that the entomofauna could play a main role as a selective pressure on the waxy or viscoelastic trapping systems, while Moran et al. (2013) suggested that those systems are more constrained by climate. Both are likely to be linked. In fact, waxy traps that are associated with more cylinder forms with narrow apertures (this study) are more abundant in lowlands associated with seasonal climates (Moran et al. 2013) richer in ants (Gunsalam 1999; Davidson et al. 2003; Luke et al. 2014) and termites (Gathorne‐Hardy 2001; Luke et al. 2014) than perhumid montane habitats. Flying insects are, comparatively, a more important component of the montane entomofauna (Collins 1980), including, primarily, flies, the major pollinators in high‐elevation alpine or perhumid regions (Kearns 1992). The funnel‐shaped traps with a wider aperture diameter such as those of *N. inermis* and *N. jamban* are found at high‐elevation sites and have a fluid with very viscous properties that catches flies and midges as the main prey (Clarke 2001; McPherson 2009). In those perhumid regions, water evaporation is less frequent and is likely to entail a lesser cost to the plant than if those phenotypes were found in the seasonally dry regions of the lowlands. This finding may explain why those funnel shapes are mostly found in the mossy forest of montanes and why waxy slender forms, often associated with a narrow aperture, are more frequent in lowland regions.

We have assembled several arguments that show how *Nepenthes* pitcher plants have evolved numerous species displaying different morphological and trapping features used to exploit a variety of different resources, including nonanimal ones. Thus, our study supports the hypothesis that this genus is a conspicuous example of adaptive radiation. However, to provide definite proof, this approach needs to be extended to other species and completed with transplantation experiments, a systematic characterization of the resources available in the environments and tests of phylogenetic constraints. Such an adaptive radiation might be comparable to the one undergone by the genus *Brocchinia*, a genus of Bromeliad growing in the tepuis of Venezuela and Guyana, which displays different nutritional strategies and tank leaf forms in response to extreme nutrient poverty (Givnish et al. 1997, 2014). During periods between glaciations, the *Nepenthes* species may have extended their distribution range, and several species may have secondarily encountered each other in the same geographic zone, being subject to strong interspecific competition for the available resources. Such strong competition, as observed in the heath forest of Borneo, may have driven the occurrence of character displacement (Beans 2014), which even more strongly favored niche diversification in this genus. *Nepenthes hemsleyana*, the pitcher form of which is adapted to harbor a small bat species (Grafe et al. 2011) and which often shares the same habitat with *N. rafflesiana,* a presumably sister species (Gaume and Di Giusto 2009), might be an example of such a secondary character displacement adapted to the exploitation of a new resource, bat feces.

## Conflict of Interest

None declared.

## Supporting information


**Table S1.** Main pitcher characteristics of the studied *Nepenthes* taxa.

**Table S2.** Estimates of pitcher volumes from pitcher dimensions.
