# Supplementary material for: Different pitcher shapes and trapping syndromes explain resource partitioning in Nepenthes species
Source: Ecol Evol. 2016 Feb 3;6(5):1378–92. doi: 10.1002/ece3.1920 (PMC4739188; doi:10.1002/ece3.1920)
Supplement: Supplementary file 1 — Table S1. Main pitcher characteristics of the studied Nepenthes taxa. [file ECE3-6-1378-s001.docx]

**Supporting information**

Table S1 Main pitcher characteristics of the studied *Nepenthes* taxa. *albo*: *Nepenthes albomarginata*, *amp*: *N. ampullaria*, *bic*: *N. bicalcarata*, *gig*: *N. rafflesiana* var. *gigantea*, *gra*: *N. gracilis*, *hems*: *N. hemsleyana*, *raf*: *N. rafflesiana* var. *typica*. Means ± SE are provided for each of the quantitative characteristics. The different letters refer to means that are significantly different from each other, as predicted by a Tukey test performed on lsmeans in the GLM, negative binomial or Poisson regression analyses.

|  |  | **Attraction features** | | | | **Capture features** | | |  | **Main quantitative characteristics** | | | | | |
| --- | --- | --- | --- | --- | --- | --- | --- | --- | --- | --- | --- | --- | --- | --- | --- |
| **species** | **pitcher type** | **dominant color** | **nectar** | **odor** | **trichome** | **waxy zone** | **fluid viscosity** | **ant symbiont** | **pitcher volume**  **(ml)** | | **height____peristome**  **(cm)** | **pitcher diameter**  **(cm)** | **fluid pH** | **prey number** | **order taxa number** |
| *albo* | lower | green | 0 | 0 | 1 | 1 | 0 | 0 | 24.3 ± 1.6 (n=86) **c** | | 7.8 ± 0.2 (n=90) **def** | 2.0 ± 0.0 (n=90) **e** | 3.5 ± 0.1 (n=30)  **b** | 1255.6±726.1 (n=5) **a** | 2.6 ± 0.2 (n=5) **d** |
|  | upper | green | 0 | 0 | 1 | 1 | 0 | 0 | 13.9 ± 1.6 (n=15) **c** | | 9.7 ± 0.3 (n=15) **d** | 1.8 ± 0.1 (n=15) **e** |  | 202.4 ± 95.7 (n=5) **b** | 3.2 ± 0.6 (n=5) **cd** |
| *amp* | lower | green | 0 | 0 | 0 | 0 | 0 | 0 | 26.9 ± 1.7 (n=123) **c** | | 4.9 ± 0.1 (n=126) **gh** | 2.3 ± 0.1 (n=126)**e** | 4.7 ± 0.0 (n=72)  **a** | 25.4 ± 15.9 (n=5) **c** | 3.6 ± 0.7 (n=5) **bcd** |
|  | upper | green | 0 | 0 | 0 | 0 | 0 | 0 | 18.9 ± 5.7 (n=6) **c** | | 4.3 ± 0.4 (n=6) **h** | 2.2 ± 0.2 (n=6) **e** |  | 12.0 ± 2.6 (n=5) **c** | 2.8 ± 0.4 (n=5) **cd** |
| *bic* | lower | red | 1 | 1 | 0 | 0 | 0 | 1 | 246.8 ± 23.9 (n=87) **b** | | 6.9 ± 0.2 (n=93) **efg** | 4.9 ± 0.1 (n=93) **b** | 4.9 ± 0.0 (n=38)  **a** | 194.8 ± 62.8 (n=5) **b** | 3.8 ± 0.4 (n=5) **bcd** |
|  | upper | yellow | 1 | 1 | 0 | 0 | 0 | 1 | 73.8 ± 17.2 (n=15) **c** | | 7.7 ± 0.7 (n=15) **def** | 3.9 ± 0.3 (n=15) **cd** |  | 674.4 ± 175.6 (n=5) **a** | 4.2 ± 0.4 (n=5) **bcd** |
| *gig* | lower | red | 1 | 1 | 0 | 0 | 1 | 0 | 503.5 ± 76.3 (n=14) **a** | | 12.9 ± 1.3 (n=8) **c** | 7.1 ± 0.6 (n=8) **a** | 3.3 ± 0.4 (n=10)  **b** | 133.4 ± 34.6 (n=5) **b** | 4.8 ± 0.7 (n=5) **bcd** |
|  | upper | yellow | 1 | 1 | 0 | 0 | 1 | 0 | 422.5 ± 102.9 (n=4) **a** | | 22.5 ± 2.9 (n=3) **a** | 7.2 ± 1.1 (n=4) **a** |  | 128.6 ± 33.1 (n=5) **b** | 6.4 ± 0.7 (n=5) **ab** |
| *gra* | lower | yellow | 1 | 0 | 0 | 1 | 0 | 0 | 10,4 ± 0.6 (n=112) **c** | | 6.2 ± 0.1 (n=115)**fgh** | 1.6 ± 0.0 (n=115)**e** | 2.2 ± 0.1 (n=70)  **d** | 201.2 ± 47.4 (n=5) **b** | 3.4 ± 0.5 (n=5) **cd** |
|  | upper | yellow | 1 | 0 | 0 | 1 | 0 | 0 | 9.6 ± 1.2 (n=34) **c** | | 6.9 ± 0.4 (n=34) **efg** | 1.8 ± 0.1 (n=34) **e** |  | 209.4 ± 48.0 (n=5) **b** | 4.2 ± 0.6 (n=5) **bcd** |
| *hems* | lower | green | 0 | 0 | 0 | 1 | 1 | 0 | 105.1 ± 21.7 (n=12) **c** | | 9.5 ± 0.6 (n=15) **d** | 3.5 ± 0.2 (n=15) **d** | 2.8 ± 0.2 (n=38)  **c** | 25.4 ± 15.6 (n=5) **c** | 3.6 ± 0.5 (n=5) **bcd** |
|  | upper | green | 0 | 0 | 0 | 1 | 1 | 0 | 75.9 ± 8.5 (n=15) **c** | | 18. ± 0.8 (n=15) **b** | 3.7 ± 0.2 (n=15)**cd** |  | 23.6 ± 8.8 (n=5) **c** | 4.2 ± 1.0 (n=5) **bcd** |
| *raf* | lower | red | 1 | 1 | 0 | 1 | 1 | 0 | 50.8 ± 10.3 (n=30) **c** | | 6.3 ± 0.3 (n=37) **fgh** | 4.1 ± 0.3 (n=37) **bc** | 2.1 ± 0.1 (n=46) **d** | 135.4 ± 34.0 (n=5) **b** | 5.6 ± 0.7 (n=5) **abc** |
|  | upper | yellow | 1 | 1 | 0 | 0 | 1 | 0 | 46.9 ± 6.8 (n=16) **c** | | 8.9 ± 0.6 (n=16) **ed** | 4.5 ± 0.2 (n=16) **bc** |  | 127.4 ± 32.9 (n=5) **b** | 7.6 ± 0.4 (n=5) **a** |

Gaume L., Bazile V., Huguin M. & Bonhomme, V. 2016 - Different pitcher shapes and trapping syndromes explain resource partitioning in *Nepenthes* species – *Ecology and Evolution*
