# Supplementary material for: Different pitcher shapes and trapping syndromes explain resource partitioning in Nepenthes species
Source: Ecol Evol. 2016 Feb 3;6(5):1378–92. doi: 10.1002/ece3.1920 (PMC4739188; doi:10.1002/ece3.1920)
Supplement: Supplementary file 2 — Table S2. Estimates of pitcher volumes from pitcher dimensions. [file ECE3-6-1378-s002.docx]

**Supporting information**

**Table S2** Estimates of pitcher volumes from pitcher dimensions. l1: height up to peristome, l2: height up to opercula; l3: aperture maximal diameter. For *N. hemsleyana* and *N. rafflesiana* var. *gigantea*, the exact volumes were always used.

| **Species-pitcher** | **Equation** | **R²** | ***F*** | **ndf** | **ddf** | ***P*** |
| --- | --- | --- | --- | --- | --- | --- |
| Albo-lower | ln(vol)=-1.53+1.90*ln(l2)+1.07*ln(l3) | 0.98 | 548.9 | 2 | 19 | <0.0001 |
| Albo-upper | ln(vol)=-1.60+1.81*ln (l2) | 0.71 | 30 | 1 | 12 | 0.0001 |
| Amp-lower | ln(vol)=-1.81+3.21*ln(l2)-0.14*ln(l3) | 0.97 | 829.7 | 2 | 52 | <0.0001 |
| Bic-lower | ln(vol)=3.54+2.75*ln(l1)+0.99*ln(l2) | 0.99 | 441.8 | 2 | 13 | <0.0001 |
| Bic-upper | ln(vol)=-2.26+1.93*ln(l1)+1.14*ln(l3) | 0.99 | 1264 | 2 | 12 | <0.0001 |
| Gra-lower | ln(vol)=-1.74+1.75*ln(l1)+1.06*ln(l3) | 0.98 | 1213 | 2 | 46 | <0.0001 |
| Gra-upper | ln(vol)=-1.22+1.13*ln(l1)+1.68*ln(l3) | 0.96 | 333.7 | 2 | 31 | <0.0001 |
| Raf-lower | ln(vol)=1.44*ln(l1)+1.06*ln(l3) | 0.98 | 641.5 | 2 | 23 | <0.0001 |
| Raf-upper | ln(vol)=-1.67+1.17*ln(l1)+1.62*ln(l3) | 0.95 | 135.4 | 2 | 13 | <0.0001 |

Gaume L., Bazile V., Huguin M. & Bonhomme, V. 2016 - Different pitcher shapes and trapping syndromes explain resource partitioning in *Nepenthes* species – *Ecology and Evolution*
